# A rare case of stiff left atrial syndrome caused by both coconut left atrium and vertebral compression: a case report

**DOI:** 10.1093/ehjcr/ytz154

**Published:** 2019-09-14

**Authors:** Junichi Ooka, Kensuke Matsumoto, Morihiko Kondo, Toshiyuki Otomo

**Affiliations:** 1 Division of Cardiovascular Medicine, Japan Community Healthcare Organization, Kobe Central Hospital, 2-1-1, Souyama-cho, kita-ku, Kobe, Japan; 2 Division of Cardiovascular Medicine, Department of Internal Medicine, Kobe University Graduate School of Medicine, 7-5-2, Kusunoki-cho, Chuo-ku, Kobe, Japan

**Keywords:** Stiff left atrial syndrome, Coconut left atrium, Heart failure, Left atrial calcification, Vertebral compression, Case report

## Abstract

**Background:**

Calcification of the left atrium (LA) is a rare condition and can be the result of long-standing rheumatic mitral stenosis or an unusual complication after mitral valve replacement. Cases of massive LA calcification have sometimes been referred to as those with ‘coconut LA’ or ‘porcelain LA’.

**Case summary:**

A 75-year-old woman was referred to our hospital because of chest discomfort and exertional dyspnoea. Doppler echocardiography revealed the presence of elevated filling pressure with significant LA dysfunction. A cardiac catheter examination revealed a quite impressive pulmonary capillary wedge pressure waveform with a steep up-slope and prominent *v* wave of 43 mmHg (mean: 15 mmHg). Multidetector row computed tomography revealed that LA was sandwiched by dense calcifications along the roof and bottom of the LA, and the posterior wall was compressed by a vertebral body. Integration of these functional and anatomical findings ultimately led to the diagnosis of ‘stiff LA syndrome’.

**Discussion:**

She had a history of tuberculosis but no history of rheumatic fever or cardiac surgery. Thus, it appeared that the tuberculous pericarditis might have led to the calcified LA by long lasting inflammation. In this case, the LA was encased by a dense calcification and compressed by vertebral body from the posterior direction. Therefore, we speculated that the LA pressure could easily elevate even with a slight haemodynamic load in this special case, and thus eventually resulting in decompensated heart failure.

For the podcast associated with this article, please visit https://academic.oup.com/ehjcr/pages/podcast

**Learning points**

- Although coconut left atrium (LA) has long been believed as the result of rheumatic heart disease or an unusual complication after mitral valve replacement, tuberculous pericarditis could be a possible aetiology.
- In stiff LA syndrome resulting from atrial calcification, the LA can no longer function as a reservoir or booster pump but merely act as a conduit.
- Giant *v* wave with steep ascending limb is an important clue for the diagnosis of stiff LA syndrome.

## Introduction

Calcification of the left atrium (LA) is a rare condition and can be the result of long-standing rheumatic mitral stenosis^1^^,^^2^ or an unusual complication after mitral valve replacement.^3^^,^^4^ Cases with massive LA calcification are referred to as those with ‘coconut LA’^5^ or ‘porcelain LA’.^3^ This condition leads to the crucial impairment of LA compliance and thus a significant increase in LA pressure, which eventually results in intractable heart failure.^6^ Here, we report a rare case with stiff LA syndrome caused by both coconut LA and vertebral compression, with no history of rheumatic heart disease or cardiac surgery.

## Timeline

**Table:** 

Time	Events
Six years ago	The patient became conscious of exertional dyspnoea and leg oedema.
Initial presentation	She came to our hospital because of the chest discomfort. Chest radiography showed bilateral pulmonary oedema and pleural effusion, which was consistent with decompensated heart failure.
Admission	Echocardiography revealed the presence of significant pulmonary hypertension and markedly reduced left atrial (LA) reservoir and booster pump function. Multidetector row computed tomography revealed that LA was encased by dense calcification and compressed by vertebral body. By cardiac catheter examination, prominent *v* wave with giant peak of 43 mmHg was observed in pulmonary capillary wedge pressure waveform. Patient was managed conservatively with loop diuretics and tolvaptan which led to amelioration of heart failure symptoms.
After discharge	Fortunately, the patient can lead her daily life without worsening of heart failure symptoms under oral administration of diuretics.

## Case presentation 

**Figure 1:**
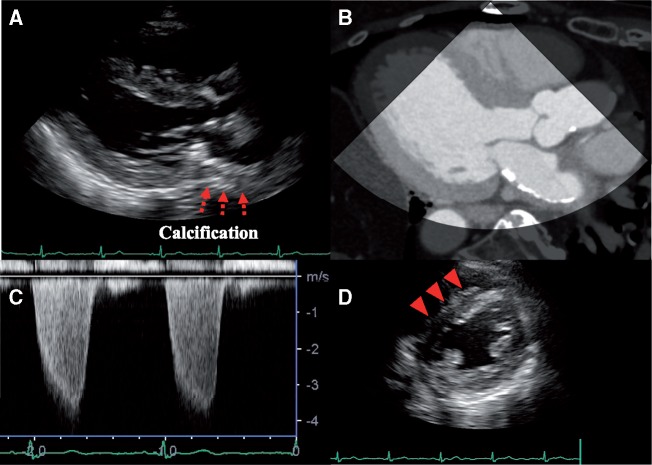
(*A*) Parasternal long-axis view showing a linear high-echoic lesion along the posterior left atrial wall (red arrows), which was demonstrated to be left atrium calcification by multidetector row computed tomography (*B*). A continuous-wave Doppler signal of tricuspid regurgitation is shown (*C*). Peak pressure gradient across the tricuspid valve was measured as 54 mmHg, and configuration of the ventricular septum (red triangle) was distorted to a D-shape in diastole (*D*), suggesting significant pulmonary hypertension.

**Figure 2:**
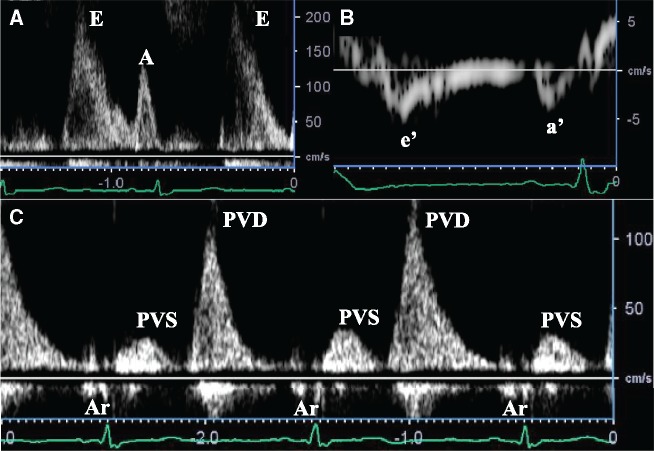
Transmitral (*A*) and mitral annular velocities (*B*) disclosed the presence of the severely elevated ventricular filling pressure (*E*/*e*' ratio of 35.5). Pulmonary venous flow showed a significantly blunted systolic forward flow and retrograde flow reversal during atrial contraction, and conversely, a prominent diastolic wave velocity was observed (*C*). Ar, pulmonary venous atrial reversal flow; PVD, pulmonary venous flow in diastole; PVS, pulmonary venous flow in systole.

**Figure 3:**
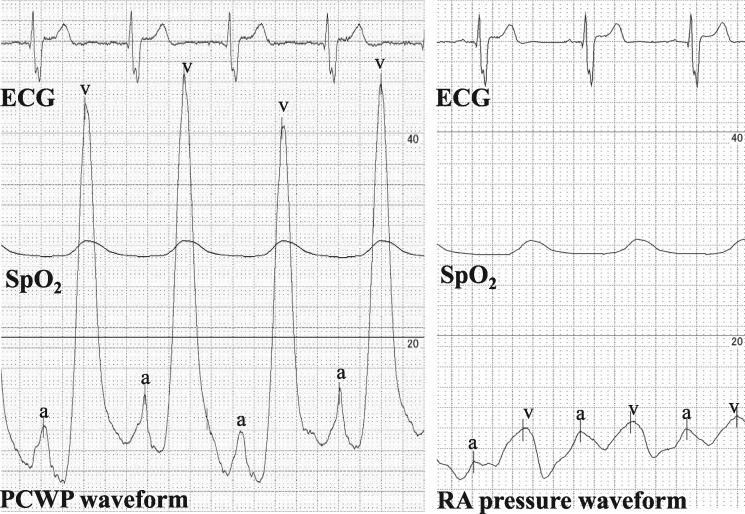
Waveforms of pulmonary capillary wedge pressure and right atrial pressures are shown. The *a* wave, *v* wave, and mean pressure were 13, 43, and 15 mmHg, respectively. A prominent *v* wave of the pulmonary capillary wedge pressure waveform was impressive. Note the marked contrast with right atrial pressure waveform. ECG, electrocardiogram; PCWP, pulmonary capillary wedge pressure; RA, right atrial; SpO_2_, saturation of percutaneous oxygen.

**Figure 4:**
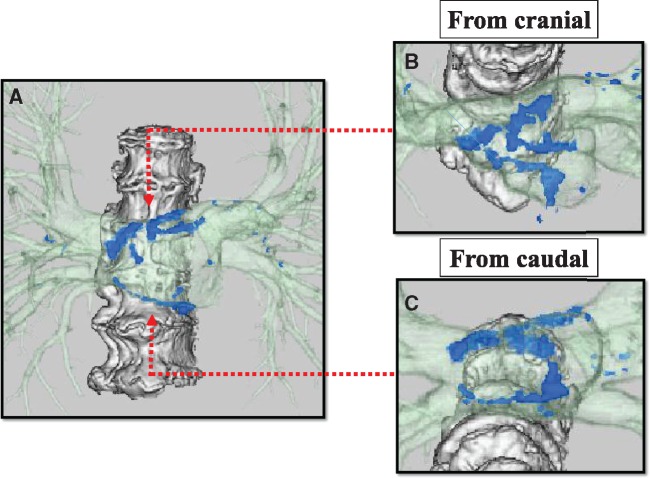
Volume-rendered images from the multidetector row computed tomography are shown (*A*). The structures coloured in light green indicate the left atrium and pulmonary veins, and blue structures represent calcium deposition. Note that the left atrium calcifications were predominantly distributed both at the roof (*B*) and bottom (*C*) of the atrial wall, thereby sandwiching the left atrium.

**Figure 5:**
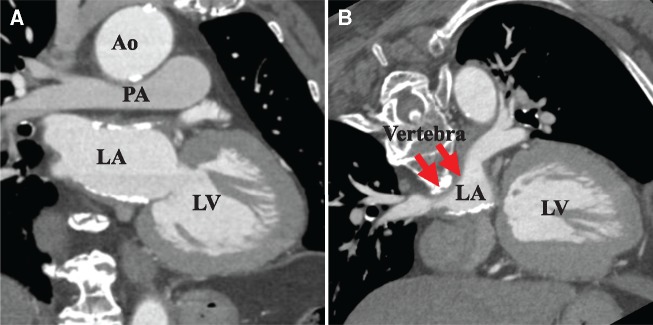
Multiplanar reconstruction images from multidetector row computed tomography are shown. The left atrium is sandwiched between dense calcification (*A*). Moreover, the posterior wall (red arrow) is compressed by the vertebral body (*B*). LA, left atrium; VB, vertebral body.

A 75-year-old woman was referred to our hospital because of chest discomfort and exertional dyspnoea. She had no remarkable medical history of rheumatic fever or cardiac surgery, except for a history of tuberculosis when she was 10 years old. On auscultation, a Grade 2/6 systolic murmur at the left lower sternal border was observed. On visual inspection, she was found to have systemic oedema and jugular distension. Electrocardiography showed sinus tachycardia with incomplete right bundle branch block and right-axis deviation. A chest radiography showed bilateral pulmonary oedema and pleural effusion, which was consistent with decompensated heart failure. Interestingly, however, laboratory findings showed a disproportionally low serum brain natriuretic peptide concentration of 63.4 pg/mL. And the qualitative analysis of Troponin T was negative at initial check-up. On the other hand, echocardiography revealed an unusual finding of the linear high-echoic lesion along the posterior LA wall, suggesting dense calcium deposition (*Figure 1A* and *B* and Supplementary material online, *Video S1*). There was moderate tricuspid regurgitation, with a peak pressure gradient of 54 mmHg, and the configuration of the ventricular septum was distorted to a D-shape in systole, which was consistent with significant pulmonary hypertension (*Figure 1C* and *D* and Supplementary material online, *Video S2*). However, there were no remarkable aortic and mitral valve dysfunctions, and her left ventricular (LV) contractile function was preserved with an ejection fraction of 78% (Supplementary material online, *Video S3*). Transmitral and mitral annular velocities disclosed the presence of severely elevated LV filling pressure with an early diastolic and atrial wave velocity (*E*/*A*) and *E*/*e*’ ratios of 1.9 and 35.5, respectively. Of note, her pulmonary venous flow showed a significantly blunted systolic forward flow (S) and retrograde flow during atrial contraction (Ar), conversely, a prominent diastolic wave (D) was observed (*Figure 2*). Despite the presence of these severe LA functional abnormalities, interestingly, her LA was not dilated at all (LA volume index of 25.5 mL/m^2^). The patient underwent cardiac catheter examination for further investigation of her strange hemodynamic state. Her pulmonary capillary wedge pressure (PCWP) waveform was impressive as shown in *Figure 3*. Especially, the morphology and the magnitude of *v* wave, which consisted of a steep ascending limb with giant peak of 43 mmHg (mean pressure of 15 mmHg), was consistent with an extremely stiff LA. Multidetector row computed tomography (MDCT) clearly revealed that the dense calcifications were predominantly distributed along the roof and bottom of the LA wall: therefore, the LA was sandwiched between the dense calcifications (*Figure 4*). Furthermore, the LA posterior wall was posteriorly compressed by the vertebral body (*Figure 5*). Integration of these functional and anatomical findings ultimately led to the diagnosis of stiff LA syndrome caused by both coconut LA and vertebral compression.

After the administration of diuretics, with azosemide 30 mg daily and tolvaptan 7.5 mg daily, her systemic oedema and heart failure symptoms gradually ameliorated, without worsening of renal function. Although this therapeutic trial was effective, her heart failure symptom was still presented in New York Heart Association II. We thus proposed surgery after medical management. However, she refused cardiac surgery and was discharged after 25 days and continued to receive close outpatient follow-ups.

## Discussion

Calcified LA is usually anecdotally reported as ‘coconut LA’^5^ or ‘porcelain LA’^3^ and has been diagnosed mostly in long-standing rheumatic mitral stenosis^1^^,^^2^ or patients with previous mitral valve replacement.^3^^,^^4^ Thus, prolonged inflammation and excessive extension of the LA wall may lead to LA calcification.^2^ In this case, however, the patient had a history of tuberculosis when she was 10 years old but no history of rheumatic fever or cardiac surgery. Therefore, we hypothesized that the tuberculous pericarditis led to the calcified LA due to long-lasting mural inflammation.

From a haemodynamical point of view, Doppler findings of the pulmonary venous flow velocities, which consisted of a significantly blunted systolic forward flow and a retrograde flow during atrial contraction as well as a prominent diastolic wave, indicated that her LA could no longer function as a reservoir or booster pump but merely act as a conduit. The PCWP waveform also clearly revealed the hemodynamic characteristics of this special case. Generally, the *v* wave on the PCWP trace reflects LA filling during ventricular systole, and thus the morphology and magnitude of the *v* wave is determined principally by the pressure–volume relationships of the LA. In a stiff LA, a given increase in LA volume during ventricular systole will produce a much larger increase in LA pressure and result in a large *v* wave. As observed in this case, the giant *v* wave with steep ascending limb was consistent with extremely stiff LA.

From an anatomical point of view, her LA was sandwiched by dense calcifications and compressed posteriorly by the vertebral body. These anatomical situations surrounding her LA may be the reason for why her LA could not be enlarged, even when her LA pressure was elevated. We speculated that her LA pressure could easily elevate, even with a slight haemodynamic load^7^ and especially while excerting, and thus can easily lead to decompensation. Especially, since her heart failure syndrome was not related to LV dysfunction but originated from a stiff LA, brain natriuretic peptide levels did not increase.

A previous report described successful management of stiff LA syndrome through surgical resection of the calcified atrial endothelium.^8^ In general, the atrial calcification does not extend beyond the atrial endocardium in patients with porcelain LA, thus the preferred surgical approach for left atrial calcification has been reported to be a total endoatrioectomy for these patients.^5^

In conclusion, we report a unique case with stiff LA syndrome caused by both coconut LA and vertebral compression. Echocardiography was a key tool that was used to initially identify this unique pathophysiology of LA, and the complementary modalities including cardiac catheter examination and MDCT were useful for a definite diagnosis. To the best of our knowledge, this is the first reported case of stiff LA syndrome caused by both coconut LA and vertebral compression that was diagnosed using multimodality imaging.

## Lead author biography 

**Figure:**
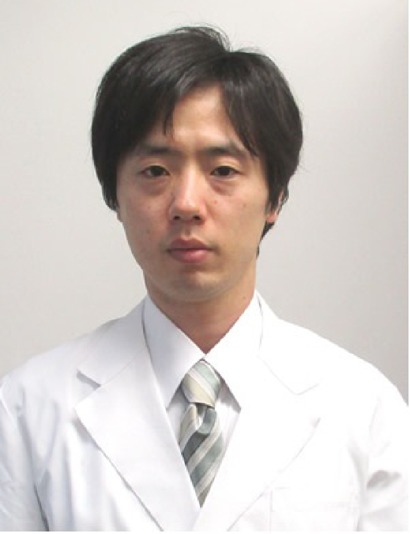


After graduating from university in 2008, Junichi Ooka went into the path of cardiology after 2 years of residency. After studying general cardiology for 3 years, he have conducted research activities focusing on echocardiography for 4 years at Kobe University.

## Supplementary Material

ytz154_Supplementary_Data
