# Supplementary material for: A rare case of stiff left atrial syndrome caused by both coconut left atrium and vertebral compression: a case report
Source: Eur Heart J Case Rep. 2019 Sep 14;3(3):ytz154. doi: 10.1093/ehjcr/ytz154 (PMC6764553; doi:10.1093/ehjcr/ytz154)
Supplement: ytz154_Supplementary_Data [file ytz154_supplementary_data.zip › ytz154-Suppl_data/Supplementary_Slide_Set.pptx]

## Slide 1
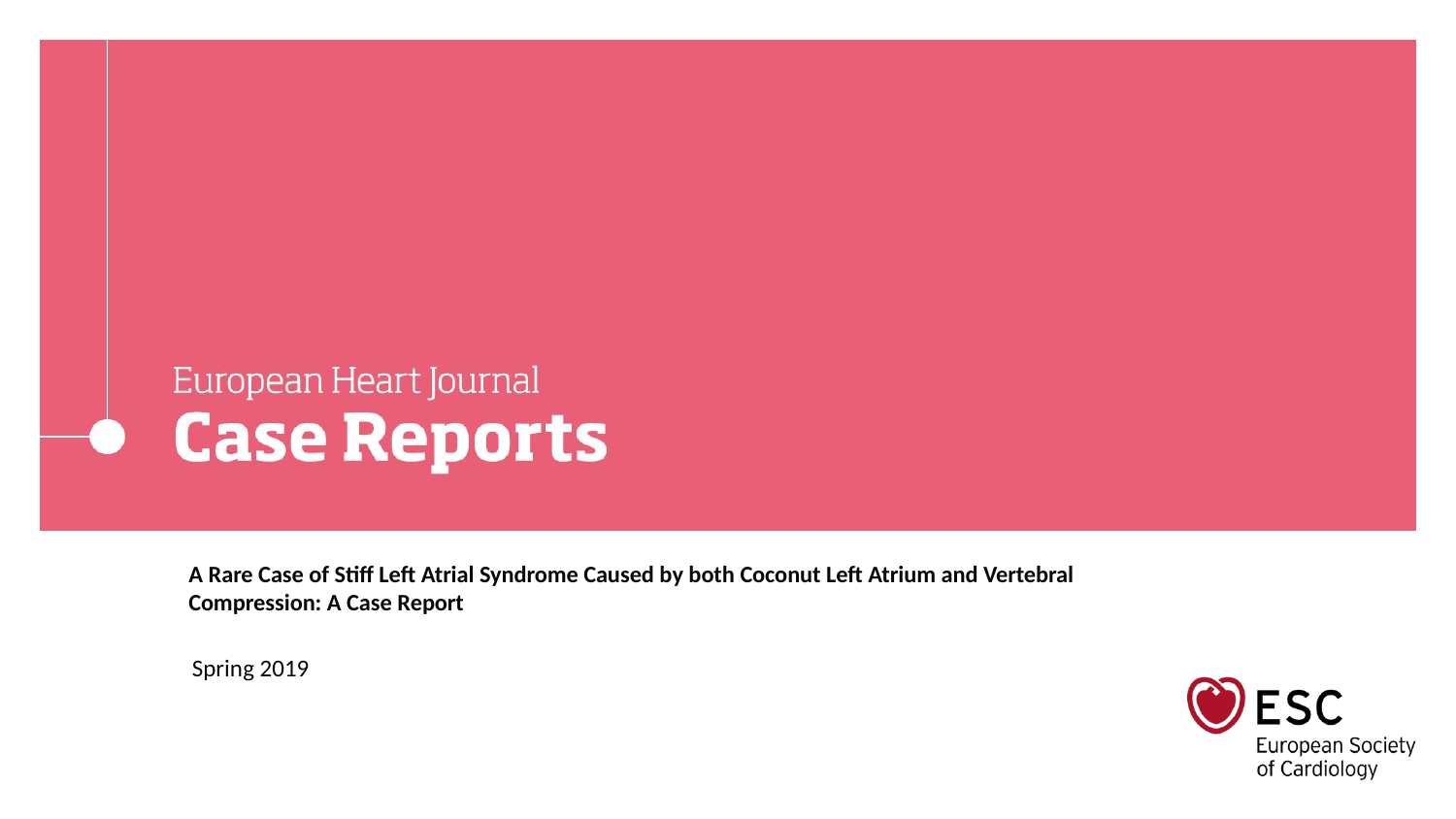

# A Rare Case of Stiff Left Atrial Syndrome Caused by both Coconut Left Atrium and Vertebral Compression: A Case Report
Spring 2019

## Slide 2
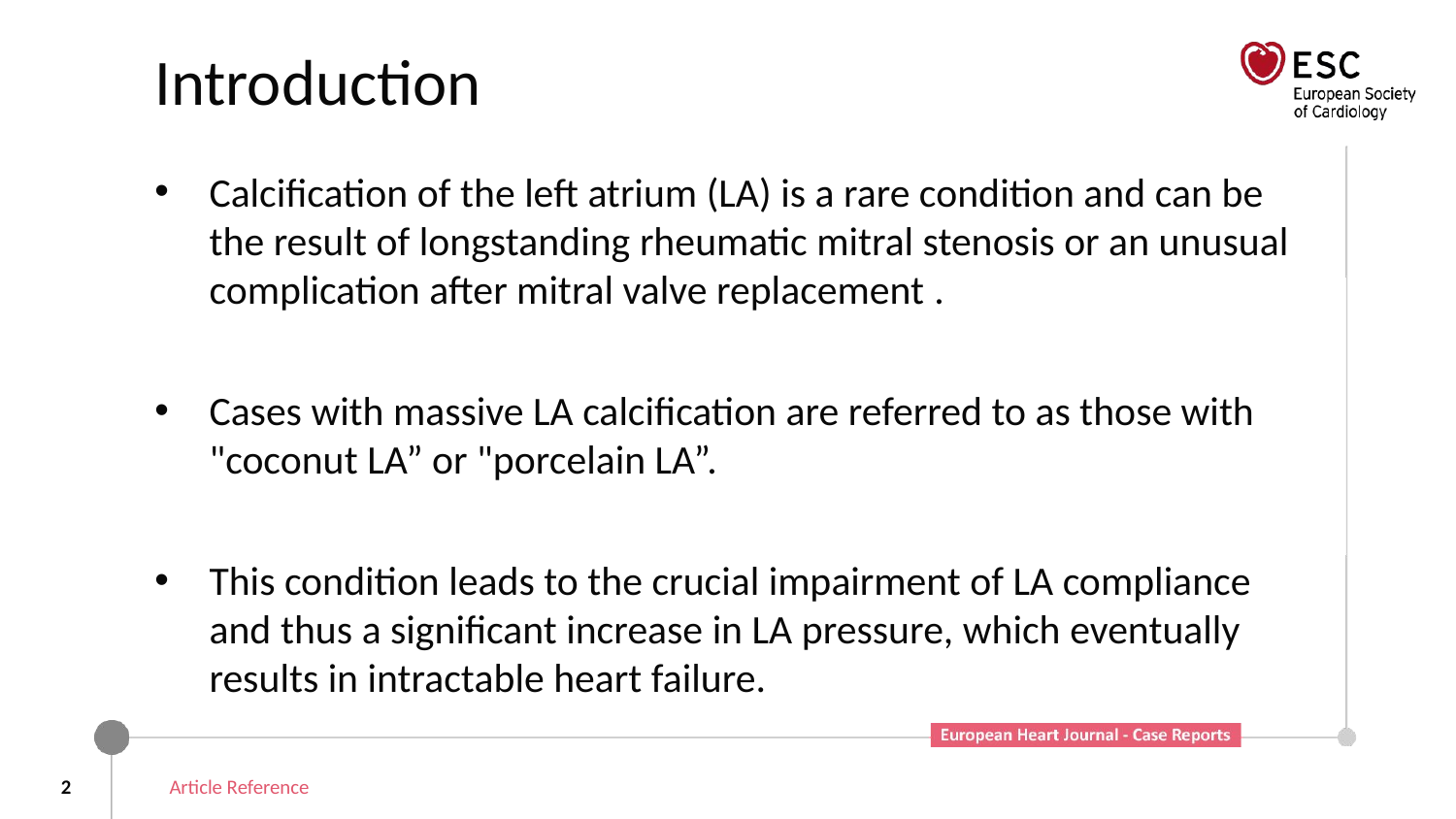

# Introduction
Calcification of the left atrium (LA) is a rare condition and can be the result of longstanding rheumatic mitral stenosis or an unusual complication after mitral valve replacement .
Cases with massive LA calcification are referred to as those with "coconut LA” or "porcelain LA”.
This condition leads to the crucial impairment of LA compliance and thus a significant increase in LA pressure, which eventually results in intractable heart failure.
2
Article Reference

## Slide 3
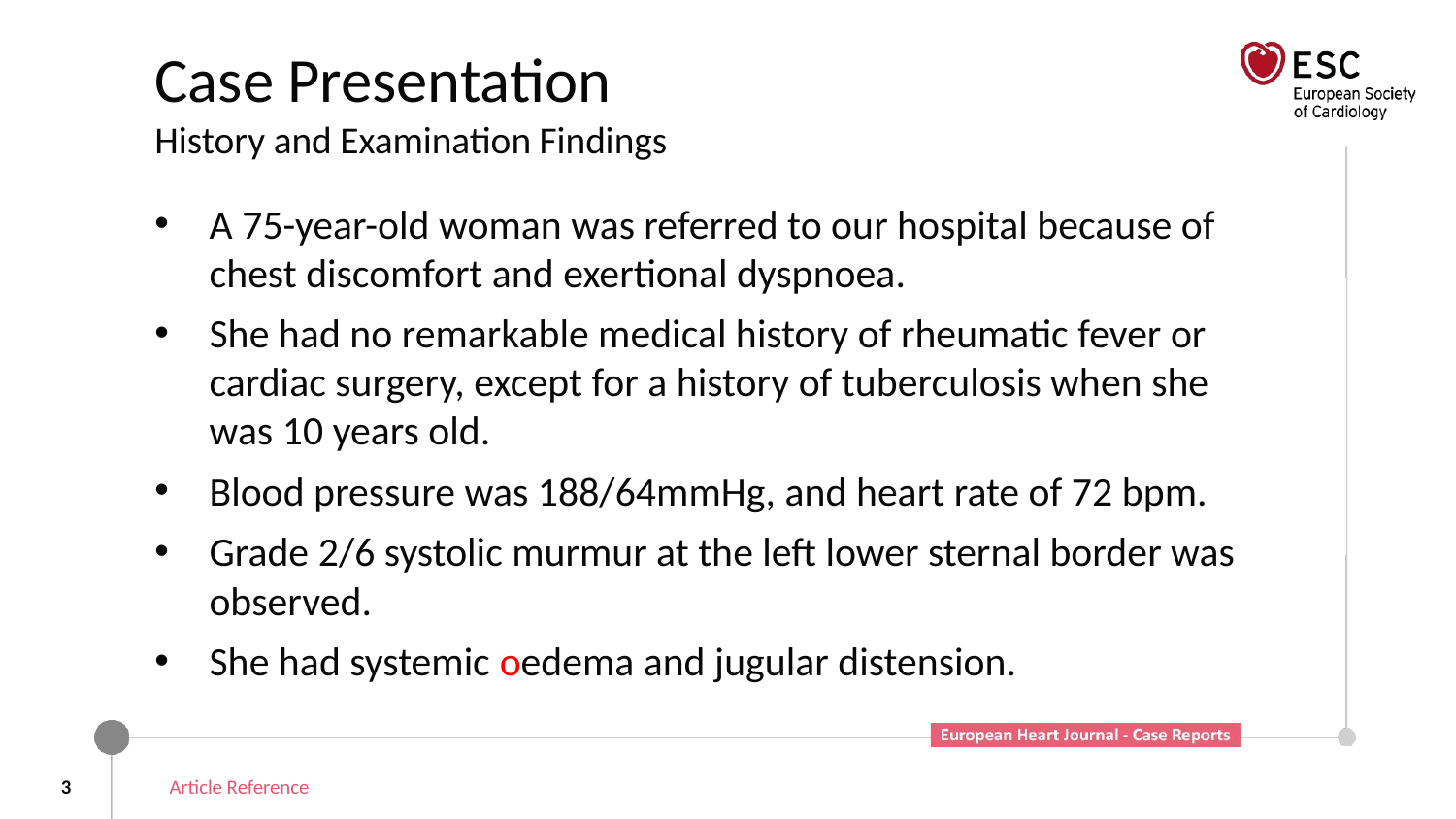

# Case Presentation History and Examination Findings
A 75-year-old woman was referred to our hospital because of chest discomfort and exertional dyspnoea.
She had no remarkable medical history of rheumatic fever or cardiac surgery, except for a history of tuberculosis when she was 10 years old.
Blood pressure was 188/64mmHg, and heart rate of 72 bpm.
Grade 2/6 systolic murmur at the left lower sternal border was observed.
She had systemic oedema and jugular distension.
3
Article Reference

## Slide 4
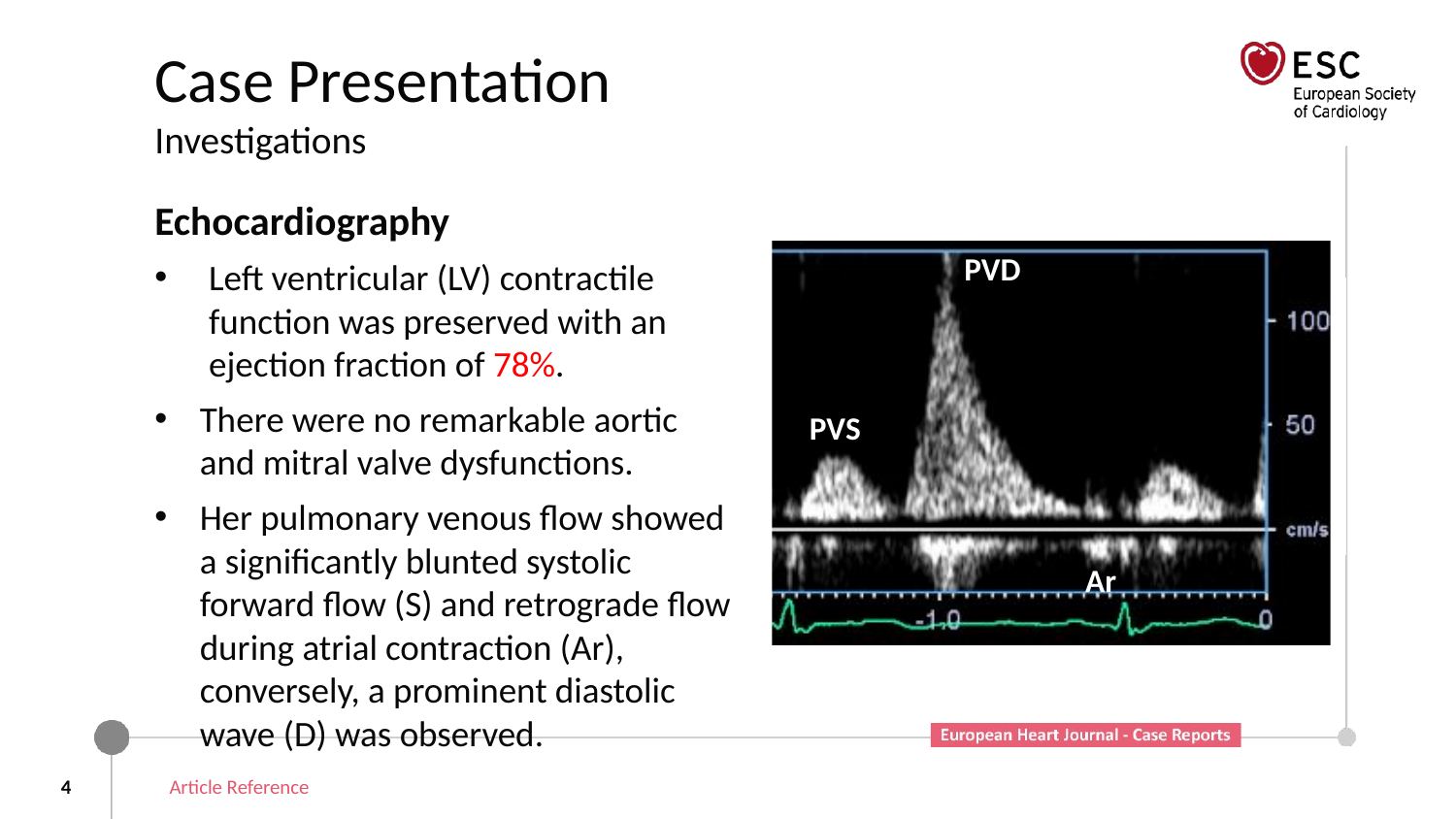

# Case Presentation Investigations
Echocardiography
Left ventricular (LV) contractile function was preserved with an ejection fraction of 78%.
There were no remarkable aortic and mitral valve dysfunctions.
Her pulmonary venous flow showed a significantly blunted systolic forward flow (S) and retrograde flow during atrial contraction (Ar), conversely, a prominent diastolic wave (D) was observed.
PVD
PVS
Ar
4
Article Reference

## Slide 5
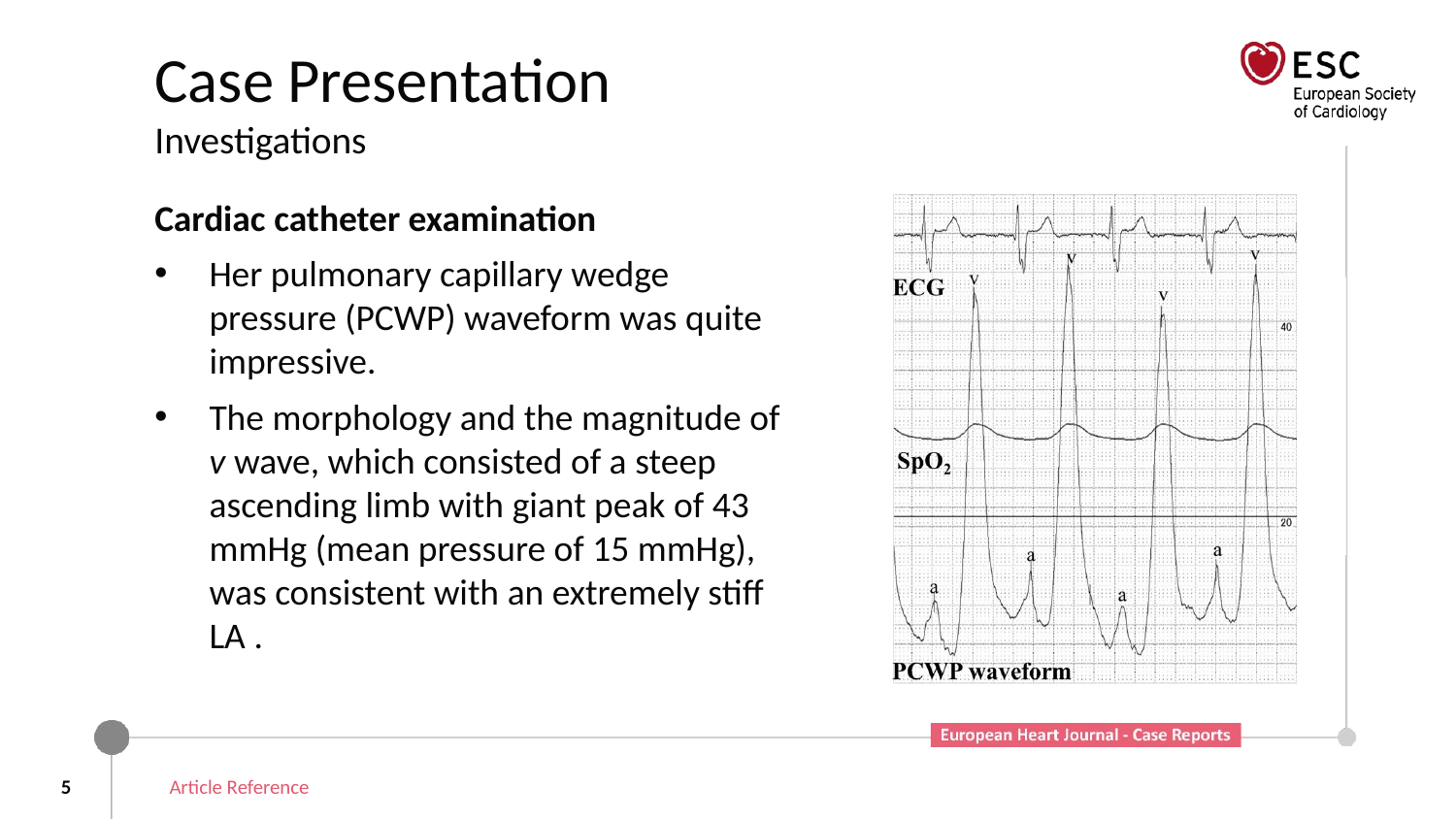

# Case Presentation Investigations
Cardiac catheter examination
Her pulmonary capillary wedge pressure (PCWP) waveform was quite impressive.
The morphology and the magnitude of v wave, which consisted of a steep ascending limb with giant peak of 43 mmHg (mean pressure of 15 mmHg), was consistent with an extremely stiff LA .
PVD
PVS
5
Article Reference

## Slide 6
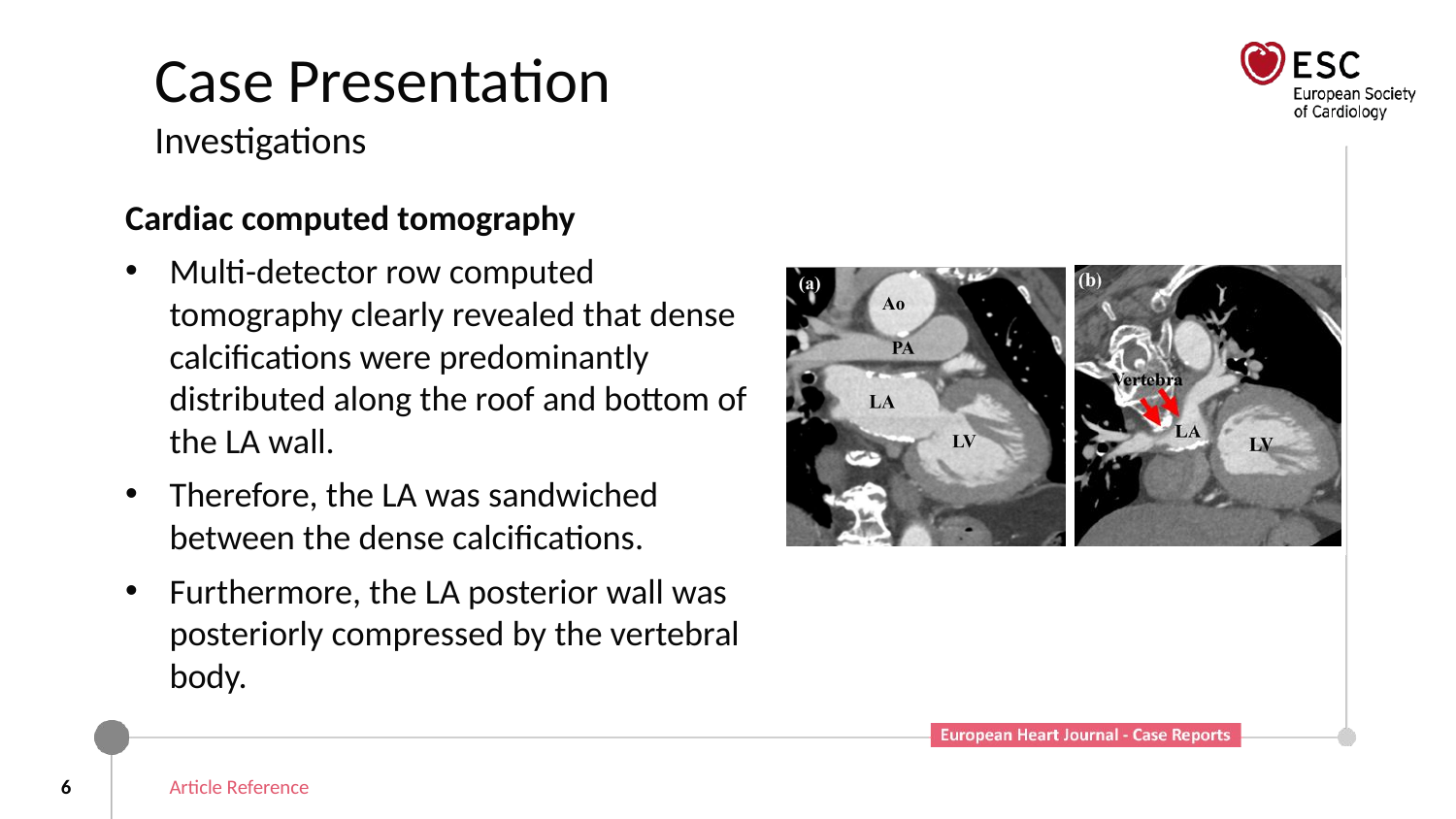

# Case Presentation Investigations
Cardiac computed tomography
Multi-detector row computed tomography clearly revealed that dense calcifications were predominantly distributed along the roof and bottom of the LA wall.
Therefore, the LA was sandwiched between the dense calcifications.
Furthermore, the LA posterior wall was posteriorly compressed by the vertebral body.
PVD
PVS
6
Article Reference

## Slide 7
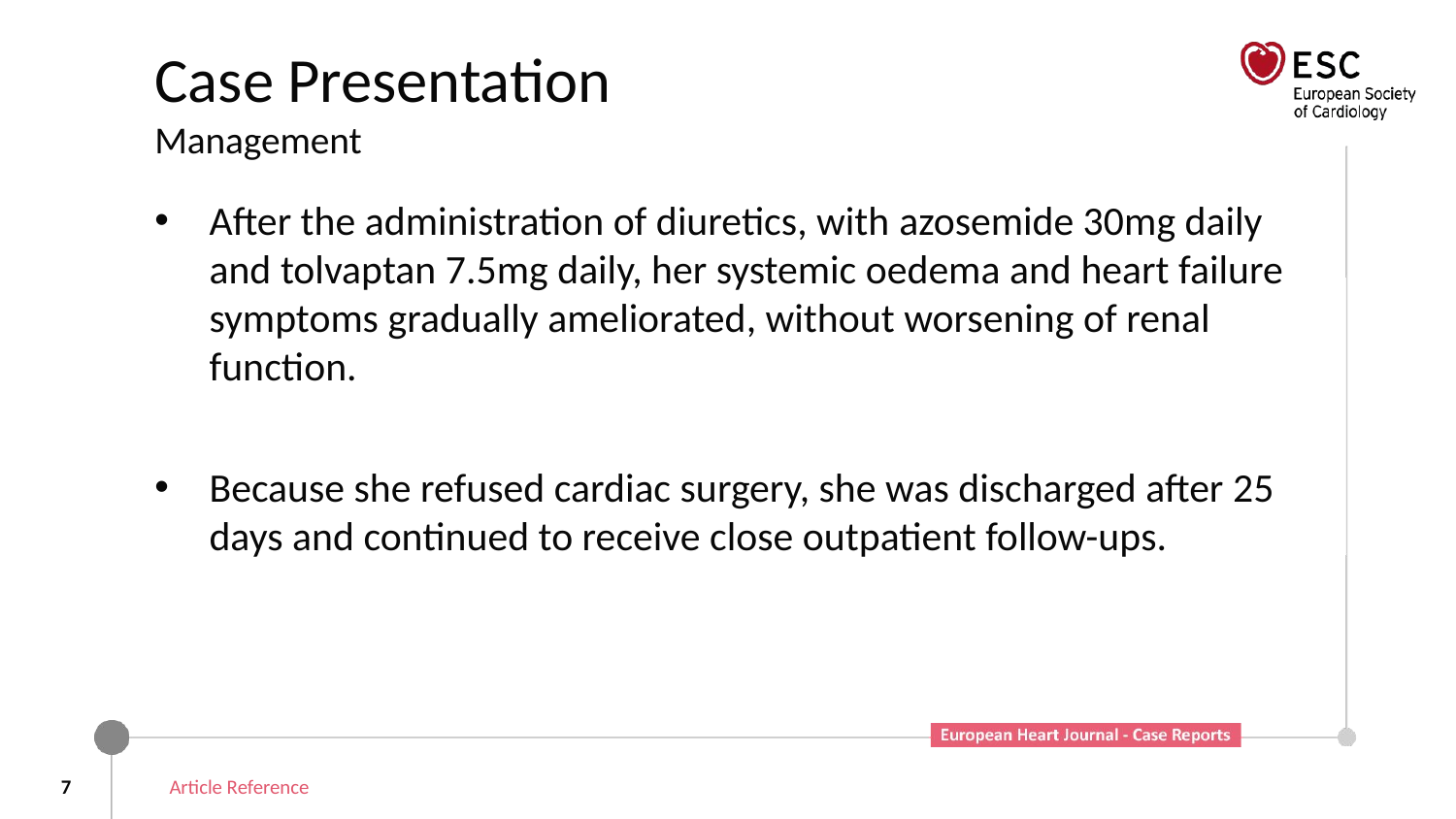

# Case Presentation Management
After the administration of diuretics, with azosemide 30mg daily and tolvaptan 7.5mg daily, her systemic oedema and heart failure symptoms gradually ameliorated, without worsening of renal function.
Because she refused cardiac surgery, she was discharged after 25 days and continued to receive close outpatient follow-ups.
7
Article Reference

## Slide 8
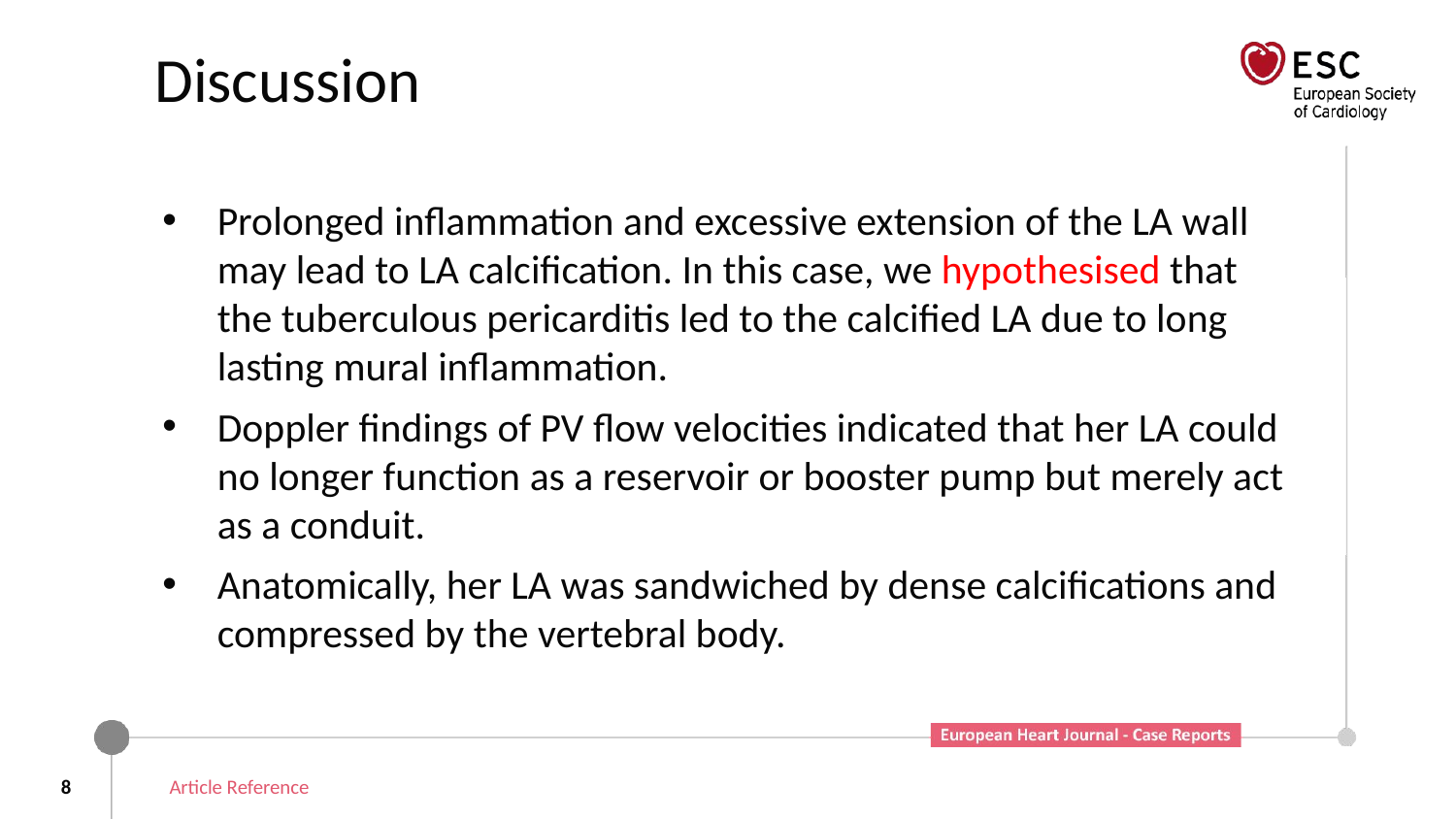

# Discussion
Prolonged inflammation and excessive extension of the LA wall may lead to LA calcification. In this case, we hypothesised that the tuberculous pericarditis led to the calcified LA due to long lasting mural inflammation.
Doppler findings of PV flow velocities indicated that her LA could no longer function as a reservoir or booster pump but merely act as a conduit.
Anatomically, her LA was sandwiched by dense calcifications and compressed by the vertebral body.
8
Article Reference

## Slide 9
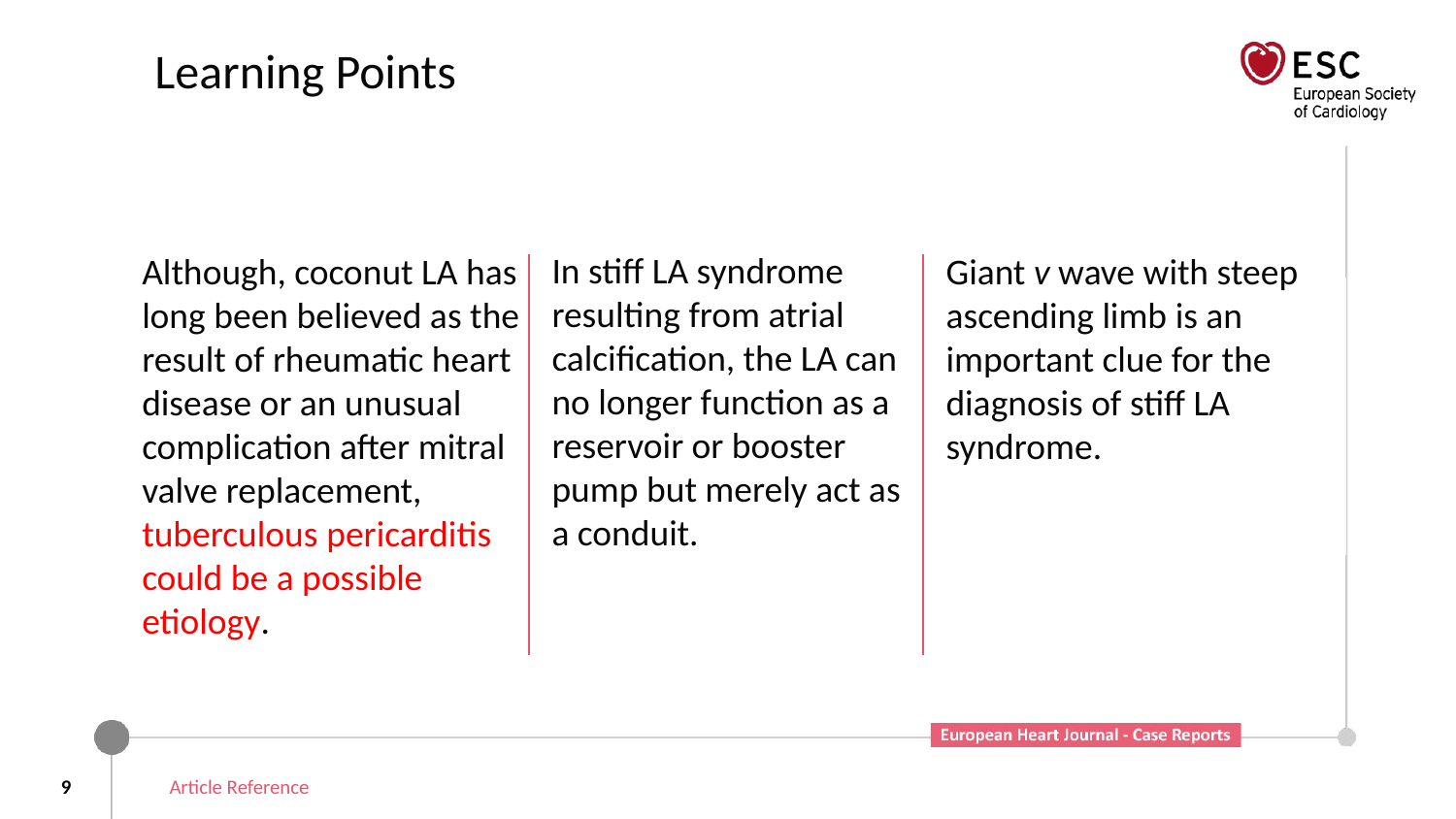

# Learning Points
In stiff LA syndrome resulting from atrial calcification, the LA can no longer function as a reservoir or booster pump but merely act as a conduit.
Although, coconut LA has long been believed as the result of rheumatic heart disease or an unusual complication after mitral valve replacement, tuberculous pericarditis could be a possible etiology.
Giant v wave with steep ascending limb is an important clue for the diagnosis of stiff LA syndrome.
9
Article Reference

## Slide 10
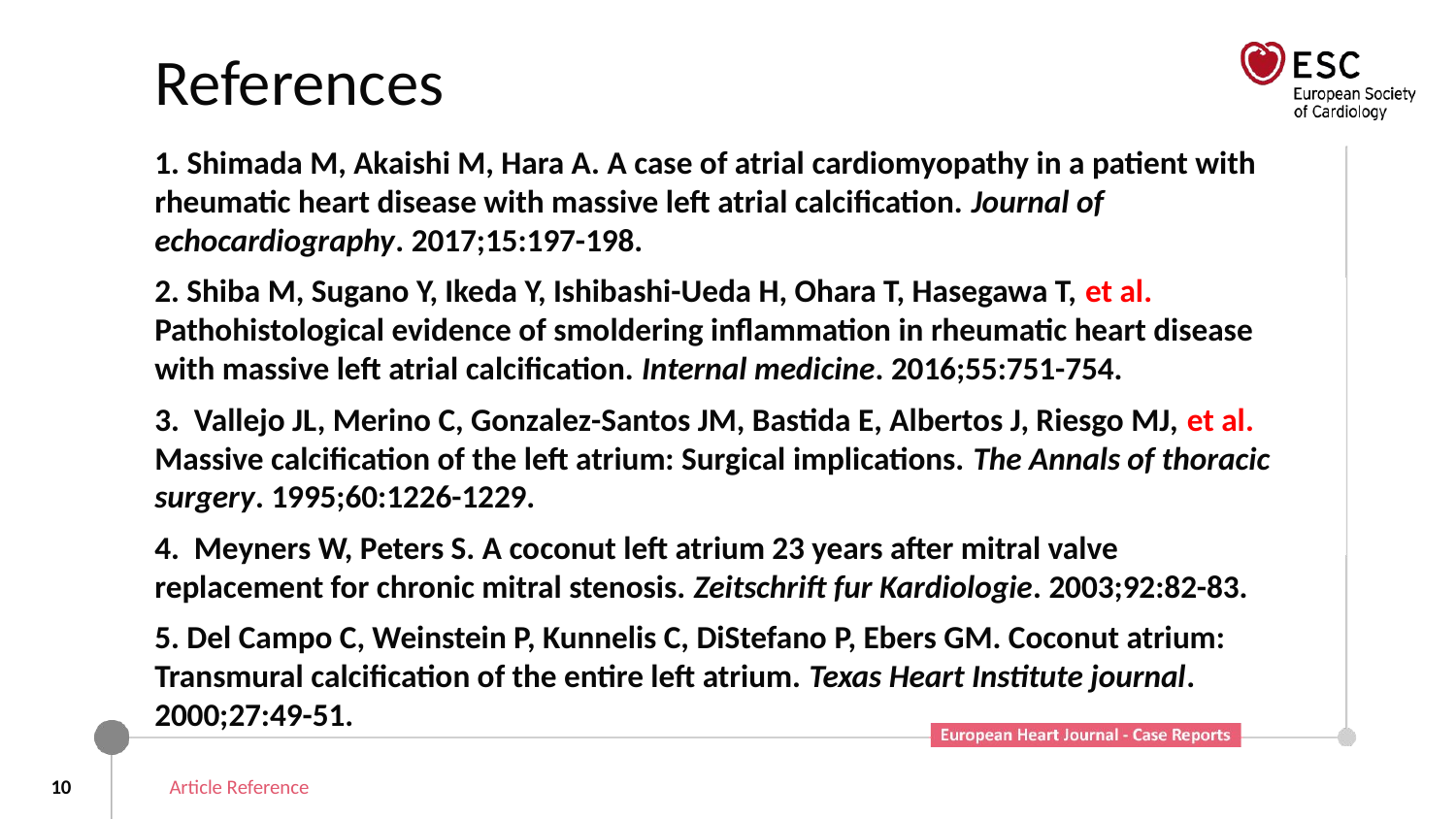

# References
1. Shimada M, Akaishi M, Hara A. A case of atrial cardiomyopathy in a patient with rheumatic heart disease with massive left atrial calcification. Journal of echocardiography. 2017;15:197-198.
2. Shiba M, Sugano Y, Ikeda Y, Ishibashi-Ueda H, Ohara T, Hasegawa T, et al. Pathohistological evidence of smoldering inflammation in rheumatic heart disease with massive left atrial calcification. Internal medicine. 2016;55:751-754.
3. Vallejo JL, Merino C, Gonzalez-Santos JM, Bastida E, Albertos J, Riesgo MJ, et al. Massive calcification of the left atrium: Surgical implications. The Annals of thoracic surgery. 1995;60:1226-1229.
4. Meyners W, Peters S. A coconut left atrium 23 years after mitral valve replacement for chronic mitral stenosis. Zeitschrift fur Kardiologie. 2003;92:82-83.
5. Del Campo C, Weinstein P, Kunnelis C, DiStefano P, Ebers GM. Coconut atrium: Transmural calcification of the entire left atrium. Texas Heart Institute journal. 2000;27:49-51.
10
Article Reference
